# Anemia prevalence, severity and associated factors among children aged 6–71 months in rural Hunan Province, China: a community-based cross-sectional study

**DOI:** 10.1186/s12889-020-09129-y

**Published:** 2020-06-23

**Authors:** Huixia Li, Juan Xiao, Minghui Liao, Guangwen Huang, Jianfei Zheng, Hua Wang, Qun Huang, Aihua Wang

**Affiliations:** 1 Department of Child Health Care, Hunan Provincial Maternal and Child Health Care Hospital, No.53, Xiangchun Road, Kaifu District, Changsha, 410008 Hunan Province China; 2 NHC Key Laboratory of Birth Defect for Research and Prevention, Hunan Provincial Maternal and Child Health Care Hospital, Changsha, Hunan Province China; 3 Department of Emergency Medicine, Second Xiangya Hospital, Central South University, Changsha, Hunan Province China; 4 Department of Maternal Health Care, Hunan Provincial Maternal and Child Health Care Hospital, No.53, Xiangchun Road, Kaifu District, Changsha, 410008 Hunan Province China; 5 Department of Information Management, Hunan Provincial Maternal and Child Health Care Hospital, Changsha, Hunan Province China

**Keywords:** Anemia, Prevalence, Severity, Children, Rural, China

## Abstract

**Background:**

In recent 10 years, with the rapid socioeconomic development and the extensive implementation of children nutrition improvement projects, the previous epidemiological data cannot reflect the actual level of anemia among children in China, especially in rural areas. Therefore, this study analyzed the prevalence, severity and associated factors of anemia among children aged 6–71 months in rural Hunan Province.

**Methods:**

A community-based cross-sectional study was conducted. Through multistage stratified cluster sampling, 5229 children aged 6 to 71 months and their caregivers were randomly selected from 72 villages across 24 towns in 12 counties from rural Hunan. The demographic characteristics of children and their caregivers, feeding practice, nutritional status of children, caregivers’ anemia-related feeding knowledge, and gestational conditions of mothers were acquired by using a unified questionnaire. Peripheral blood from the left-hand middle fingertip was sampled from each child, and hemoglobin concentration was measured using a HemoCue301 portable hemoglobin analyzer (Sweden). Associated factors analyses involving overall anemia and anemia severities were conducted on multivariate logistic regression models.

**Results:**

The overall anemia prevalence was 8.8%, and the prevalence of mild, moderate and severe anemia was 6.3, 2.5 and 0.1%, respectively. Children age groups of 6–11 months, 12–23 months and 36–47 months, exclusive breast-feeding within 6 months after birth, and maternal moderate/severe anemia were significantly associated with an increased risk of overall anemia in children. Children age groups of 6–11 months and 12–23 months were significantly associated with an increased risk of mild anemia in children. Children age groups of 6–11 months, 12–23 months and 36–47 months, low caregivers’ anemia-related feeding knowledge level, and maternal moderate/severe anemia were significantly associated with an increased risk of moderate/severe anemia in children. Children who underwent regular physical examination were less likely to have moderate/severe anemia. The common protective factor for overall, mild and moderate/severe anemia in children was high family income.

**Conclusions:**

The anemia status of preschool children in rural Hunan Province was a mild public health problem and associated with children age group, feeding practice, regular physical examination, family income, caregivers’ anemia-related feeding knowledge level, and maternal moderate/severe anemia.

## Background

Anemia is defined as the below-normal red blood cell count or hemoglobin level per unit volume in peripheral blood. Children anemia is a public health problem worldwide. Data from World Health Organization (WHO) showed that the anemia prevalence among children aged 6–59 months was 42.6% globally in 2011, and maximized in Africa (62.3%) and Southeast Asia (53.8%) [1]. The anemia prevalence of children under 5 years old in China was 12.6% and differed geographically, as it was higher in rural areas (13.3%) than in urban areas (10.3%) [2].

The causes of anemia are multifactorial, including the shortage of hematopoietic materials (e.g. iron, folic acid, vitamin A or B12), infectious diseases (e.g. malaria) and inherited hemoglobin diseases [3]. Iron deficiency is a common cause of anemia in children and is associated with 86–93% of anemia in children [4]. Many observational studies indicate that the independent risk factors of anemia in children include preterm birth, low birth weight, and maternal anemia [5–9]. Moreover, childhood anemia is closely related with feeding practice, complementary food introduction, social demographic characteristics, and family income [10–14].

Anemia epidemiological investigation among children under 5 years old was already conducted in 2010 in China to clarify the anemia prevalence among children at different age groups and in different regions [2]. However, the nutrition status of children has been significantly improved along with the rapid socioeconomic development in China and the extensive implementation of children nutrition improvement projects in rural China in recent 10 years [15, 16]. Hence, the data from the 2010 epidemiological investigation already cannot reflect the actual level of anemia among children under 5 years old in China, especially in rural areas. Recently, some children anemia status investigations have been conducted in rural China, but the investigated age ranges are narrow and concentrated on children under 2 years old, in particular, relevant data on anemic severity are insufficient [5, 7, 17–19].

Childhood anemia will adversely affect the health of children, including physical development, and may cause irreversible damages to motion, cognitional and behavioral development [3, 20, 21]. The age from 0 to 6 years old is the critical period for children’s growth and development, and it is also a high age group for anemia. Owing to limitations in socioeconomic conditions and the feeding skills of caregivers, rural children are more susceptible to anemia. Hence, systematical research about the associated factors on anemia in the whole age range of 0–6 years is critical for the prevention and treatment of children anemia in rural areas. In this study, a community-based cross-sectional study was conducted to clarify the status, severity and associated factors of anemia among children aged 6–71 months in rural Hunan Province, and to propose some appropriate intervention measures for government to further improve the anemia status of rural children.

## Materials and methods

### Subjects

The subjects were children under 6 years old (aged 6–71 months) and their caregivers from rural Hunan Province between August and November 2019. The sample size was determined according to relevant equations for cross-sectional studies [22]. Since the anemia prevalence among children in Hunan was estimated to be 9.1% [23], the size of a test α was 0.05, permissible error *d* was 0.13, the design effect of complex samples was 2 and the non-response rate was 20%, the final sample size was calculated to be 5500 (=2270 × 2 × 1.2).

Subjects were selected by multistage stratified cluster sampling. The 14 cities in Hunan were divided by the economic condition into three levels: high, moderate and low. Then 2 cities from each economic level, 2 counties from each selected city, 2 towns from each selected county, and 3 villages from each selected town were randomly selected. From each village, all eligible children and their caregivers were included into our study. Totally, 5229 children from 72 villages across 24 towns in 12 counties were involved. The inclusion criteria for children were as follows: (1) residence in the study area for at least 6 months; (2) no acute or chronic diseases 1 month before the investigation; (3) no drug treatment 1 month before the investigation.

### Data collection

This study consisted of a questionnaire survey and a peripheral hemoglobin detection. The questionnaire covered 3 aspects: (1) demographic characteristics of children (gender, age, ethnicity, birth weight, preterm birth, left behind child), feeding practice (feeding practice with 6 months after birth, nutrient supplements), passive smoking, and regular physical examination, current nutritional status; (2) demographic characteristics of caregivers (type, education level, occupation) and knowledge of anemia-related feeding; (3) gestational conditions of the mothers (moderate/severe anemia, pregnancy comorbidity, folate supplementation, iron supplementation). Peripheral blood hemoglobin concentration was detected using a HemoCue301 portable hemoglobin analyzer (Sweden).

#### Definition of variables

##### Characteristics of children

Hunan is a multiracial province in central south China and is lived by 56 ethnicities, including Han, Tujia, Miao, Yao, Dong, Bai, Hui, Zhuang, etc. Han accounts for about 90% of the population in Hunan, and the other 55 ethnicities only account for 10% and are named minorities. Therefore, ethnicity was divided into Han and minorities (the other 55 ethnicities in Hunan except Han). The birth weight < 2500 g, 2500–3999 g, and ≥ 4000 g were considered as low birth weight, normal birthweight, and macrosomia, respectively. Birth < 37 gestational weeks was regarded as premature birth. Left behind children referred to those children whose mothers or/and fathers left home for work and not live with them. The feeding practice within 6 months after birth was divided into exclusive breast-feeding, predominant breast-feeding, mixed feeding, and formula feeding. Exclusive breast-feeding meant the infant only fed on breast milk and did not eat any other food or beverage or even water, but can be fed with drugs, vitamins or minerals under medical indications. Predominant breast-feeding meant the infant mainly fed on breast milk, but also took other liquids, including water, juice, dropping liquid and syrup. Mixed feeding meant the infant was fed with both breast milk and other milk and milk replacements. Formula feeding meant the infant was not fed with breast milk, but only with other milk and milk replacements. Children’s nutrient supplements included vitamin A, vitamin D, calcium, iron, zinc and DHA, and we investigated whether the child was supplemented one or more of the above nutrient agents in the past 1 week. Passive smoking meant a nonsmoker inhaled at least 15 min every day the smoke exhaled by smokers for at least 1 day within 1 week.

##### Nutritional status of children

The indicators of children’s nutritional status in this study included stunting, underweight, wasting and overweight/obesity, which were acquired by anthropometric measurements (length/height and weight) and their corresponding assessments using WHO Child Growth Standards. Children length/height and weight were measured using standardized methods described by the *Technical Specification for Children Health Check Service* (China Ministry of Health, 2012). Length of children aged 6–23 months was measured using an infant scale with accuracy of 0.1 cm; height was measured using a stadiometer for children of 24–71 months with accuracy of 0.1 cm. Body weight was measured using electric scale for children with accuracy of 0.05 kg. Length/height for age z score (HAZ), weight for age z score (WAZ), weight for length/height z score (WHZ) and body mass index (BMI) for age z score (BMIZ) were calculated with WHO anthropometric macros in SPSS (igrowup_SPSS and WHO2007_SPSS) and WHO Child Growth Standards (WHO, 2006 and 2007) [24, 25]. BMI was calculated using the ratio between children’s weight in kilograms and length/height in meters squared (kg/m^2^). HAZ < -2 was defined as stunting, WAZ < -2 was defined as underweight. For children aged 6–60 months, WHZ < -2 was defined as wasting, WHZ > 2 was defined as overweight/obesity; and for children aged 61–71 months, BMIZ <-2 was defined as wasting, BMIZ > 2 was defined as overweight/obesity.

##### Characteristics of caregivers

Caregivers were those who took care of the diets, living and personal security for children and were divided into two types: parents, and grandparents/others. The caregivers’ education level was classified into primary school or below, junior high school, senior high school, college or above. The occupation of caregivers was divided into housework, government agencies staff, business service staff, farmer, others. The knowledge of anemia-related feeding was acquired according to the following 4 open questions concerning anemia prevention in children, including (1) which nutrient deficiency is related to anemia; (2) what is the most suitable food for iron supplement for children; (3) what is the most suitable supplementary food to be first added for infants; (4) whether eating more vegetables and fruits can promote iron absorption. The corresponding correct answers for these 4 questions are iron, animal blood or red meat (such as pork, beef, mutton and so on), cereal paste foods fortified with iron, and eating more vegetables and fruits can promote iron absorption, respectively. According to their answers to the questions of anemia prevention, the anemia-related feeding knowledge level of caregivers was divided into 3 groups: a high-level group (with all 4 answers correct); a moderate-level group (with 3 answers correct); a low-level group (with 2 or less answers correct).

##### Gestational conditions of mothers

Maternal moderate/severe anemia was defined as a hemoglobin level < 100 g/L [26]. The maternal hemoglobin concentration in this study was the concentration in the third trimester of pregnancy, which was obtained based on the participants’ recall for their hemoglobin detection during pregnancy. The maternal pregnancy comorbidities included gestational diabetes mellitus, hypertensive disorders complicating pregnancy, cardiac diseases complicating pregnancy, liver diseases complicating pregnancy, and thyroid dysfunction.

#### Detection of peripheral blood hemoglobin in children

Peripheral blood was collected from the left-hand middle fingertip of each child by using exclusive reagents (blood spots). After the side of the left-hand middle fingertip was punctured using a puncture needle, the first and second drops of blood were discarded, and blood (~ 10 μl) was collected since the third drop. Then the extra blood on the front, back and bottom sides of each blood spot was cleaned off using clean paper-towel. The blood spot was put onto a HemoCue301 portable hemoglobin analyzer (Hemocue, Inc., Ängelholm, Sweden) for hemoglobin concentration detection.

#### Anemia diagnostic criteria in children

According to WHO criteria, for children aged 6–59 months, anemia is defined at Hb < 110 g/L (100–109, 70–99, and < 70 g/L correspond to mild, moderate and severe anemia, respectively). For children aged 5–11 years old, anemia is defined as Hb < 115 g/L, in which 110–114, 80–109, and < 80 g/L correspond to mild, moderate and severe anemia, respectively [26]. Hemoglobin (Hb) concentrations of children living at different altitudes were calibrated using Chinese anemia screening standard [27], except at altitude < 1000 m. An additional file shows the corrected values of Hb at different altitudes (see Additional file 1). The altitude of Hunan is within the range of 24–2042 m, and the maximum corrected value for Hb is 8 g/L.

### Quality control

The questionnaire survey was completed by child health care doctors, who conducted a face-to-face inquiry survey for the caregivers. Hb concentration was measured by technicians. Prior to the survey, the whole staffs involved were trained unifiedly, and only the qualified ones were allowed to take part in on-site investigations. During the survey, all copies of the questionnaire were checked by quality control personnel: each copy should be filled in in a complete and standard way. Any unlogical or missed response should be corrected in time. Data were double-inputted on Epidata 3.1(Jens M.Lauritsen, Michael Bruus and Mark Myatt, Odense, Denmark) and tested in terms of consistency. For any inconsistent data, the original copy should be checked to ensure the high accuracy of any inputted data.

### Statistical analysis

Statistical analyses were conducted on SPSS 25.0 (IBM, Chicago, IL, USA). The overall anemia prevalence and anemia severities among children with different characteristics were compared using chi-square test. The significant variables identified from univariate analyses were involved into multivariate logistic regression models of overall anemia and anemia severities. There were two dependent variables in the multivariate logistic regression analyses: overall anemia, and anemia severity. Overall anemia was a binary variable and was coded as 0 = no, and 1 = yes. Anemia severity was an ordinal categorical variable and was coded as 1 = no anemia, 2 = mild anemia, and 3 = moderate/severe anemia. The strength of association between significant factors and anemia was evaluated by using odds ratios (ORs) with 95% confidence interval (CI). Considering the complex design of the survey, both simple samples and complex samples statistical analyses were used in the description of results and logistic regression. All statistical tests were two-tailed, and the significant level was *P* < 0.05.

### Ethics approval and consent to participate

The study protocol was approved by the Ethics Committee of Hunan Provincial Maternal and Child Health Care Hospital (No.2019-S036). The study was conducted in accordance with the Declaration of Helsinki. Written informed consents were obtained from all the caregivers of children involved in this study.

## Results

### Characteristics of children

A total of 5500 children and their caregivers were investigated. After excluding 271 children for incomplete core information (e.g. gender, age, hemoglobin concentration), and the final sample size for analysis was 5229. As shown in Table 1, of the children investigated, 51.6% were boys and 48.4% were girls. The major age groups of children were 36–47 and 48–59 months, which accounted for 23.6 and 22.3% respectively. Most of the children were in Han ethnic group (86.1%). The proportions of low birth weight, preterm birth, and left-behind children were 3.3, 4.2 and 55.5% respectively. The proportion of exclusive breast-feeding within 6 months after birth was 47.9%; and the proportion of nutrient supplements in the past 1 week was 43.1%. 42.8% of the children were exposed to passive smoking, and 85.6% of the children underwent regular physical examinations. For the nutritional status of children, 4.8% suffered from stunting, 4.3% suffered from underweight, 5.0% suffered from wasting, and 11.6% suffered from overweight/obesity. The proportion of family annual income ≤30,000 RMB was 54.9%.

**Table 1:** Characteristics of children aged 6–71 months

Characteristics	Frequency (*n*)	Crude proportion (%) ^a^	Corrected proportion (%) ^b^
Gender of children			
Girls	2587	49.5	48.4
Boys	2642	50.5	51.6
Age of children (months)			
6–11	416	8.0	7.7
12–23	800	15.3	14.7
24–35	835	16.0	15.1
36–47	1114	21.3	23.6
48–59	1150	22.0	22.3
60–71	914	17.5	16.5
Ethnicity of children			
Han	4665	89.2	86.1
Minorities	564	10.8	13.9
Birth weight			
< 2500 g	190	3.6	3.3
2500–3999 g	4695	89.8	90.1
≥ 4000 g	344	6.6	6.6
Preterm birth			
No	4980	95.2	95.8
Yes	249	4.8	4.2
Left-behind children			
No	2257	43.2	44.5
Yes	2972	56.8	55.5
Feeding practice within 6 months after birth			
Exclusive breast-feeding	2796	53.5	47.9
Predominant breast-feeding	613	11.7	15.1
Mixed feeding	1270	24.3	25.0
Formula feeding	550	10.5	12.0
Nutrient supplements			
No	2730	52.2	56.9
Yes	2499	47.8	43.1
Passive smoking			
No	2885	55.2	57.2
Yes	2344	44.8	42.8
Regular physical examination			
No	593	11.3	14.4
Yes	4636	88.7	85.6
Stunting			
No	4994	95.5	95.2
Yes	235	4.5	4.8
Underweight			
No	5020	96.0	95.7
Yes	206	4.0	4.3
Wasting			
No	5016	95.9	95.0
Yes	213	4.1	5.0
Overweight/obesity			
No	4631	88.6	88.4
Yes	598	11.4	11.6
Family income (RMB/year)			
≤ 30,000	3218	61.5	54.9
30,001–59,999	1209	23.1	25.7
≥ 60,000	802	15.3	19.4

^a^ The proportion was calculated by simple samples analyses

^b^ The proportion was calculated by complex samples analyses

### Characteristics of caregivers

As shown in Table 2, the caregivers were mostly parents (65.4%). Most of the caregivers had junior middle school education (39.8%), but the proportion of primary school or below was as high as 22.8%. The dominant occupation was housework (58.6%). The proportions of caregivers with low-, moderate- and high-level of anemia-related feeding knowledge were 26.6, 34.2 and 39.3%, respectively.

**Table 2:** Characteristics of children’s caregivers

Characteristics	Frequency (*n*)	Crude proportion (%) ^a^	Corrected proportion (%) ^b^
Type of caregivers			
Parents	3431	65.6	65.4
Grandparents/ other	1798	34.4	34.6
Education level of caregivers			
Primary school or below	1100	21.0	22.8
Junior high school	2013	38.5	39.8
Senior high school	1178	22.5	20.4
College or above	938	17.9	17.1
Occupation of caregivers			
Housework	3023	57.8	58.6
Government agencies staff	612	11.7	11.4
Business services staff	435	8.3	7.2
Farmer	347	6.6	5.8
Other	812	15.5	17.0
Anemia-related feeding knowledge level			
Low	1365	26.1	26.6
Moderate	1827	34.9	34.2
High	2037	39.0	39.3

^a^ The proportion was calculated by simple samples analyses

^b^ The proportion was calculated by complex samples analyses

### Gestational conditions of mothers

Of the mothers during pregnancy, about 4.3% suffered from moderate/severe anemia, 7.8% suffered from pregnancy comorbidities, 87.4% were supplemented with folate, and 60.0% were supplemented with iron agents (Table 3).

**Table 3:** Gestational conditions of mothers

Characteristics	Frequency (*n*)	Crude proportion (%) ^a^	Corrected proportion (%) ^b^
Maternal moderate/severe anemia			
No	4980	95.2	95.7
Yes	249	4.8	4.3
Pregnancy comorbidity			
No	4743	90.7	92.2
Yes	486	9.3	7.8
Folate supplementation during pregnancy			
No	562	10.7	12.6
Yes	4667	89.3	87.4
Iron supplementation during pregnancy			
No	1814	34.7	40.0
Yes	3415	65.3	60.0

^a^ The proportion was calculated by simple samples analyses

^b^ The proportion was calculated by complex samples analyses

### Prevalence and severity of anemia in children

The overall anemia prevalence was 8.8%, and the prevalence of mild, moderate and severe anemia was 6.3, 2.5 and 0.1%, respectively. The overall anemia prevalence in age group of 6–11 months was highest among all age groups (22.3%), and the prevalence of mild and moderate/severe anemia was 14.9 and 7.5%, respectively. Of the children whose mothers suffered from moderate/severe anemia during pregnancy, the anemia prevalence was 13.9%, and the prevalence of mild and moderate/severe anemia was 8.7 and 5.2%, respectively. Univariate analyses showed that the overall anemia was associated with 7 factors, including children age group, feeding method within 6 months after birth, family income, education level of caregivers, caregivers’ anemia-related feeding knowledge level, maternal moderate/severe anemia during pregnancy, and pregnancy comorbidities (Table 4). The anemia severities of children were associated with 6 factors, including children age group, regular physical examination, family income, education level of caregivers, caregivers’ anemia-related feeding knowledge level, and maternal moderate/severe during pregnancy.

**Table 4:** The prevalence and severity of anemia according to children and caregivers’ characteristics

Characteristics	Frequency	Anemic status and severity level, *n* (prevalence, %)										
		Mild			Moderate/severe			*P*^c^	Overall			*P*^c^
		*n*	% ^a^	% ^b^	*n*	% ^a^	% ^b^		*n*	% ^a^	% ^b^	
**Characteristics of children**												
Gender of children								0.920				0.833
Girls	2587	168	6.5	6.2	70	2.7	2.6		238	9.2	8.8	
Boys	2642	181	6.9	6.3	87	3.3	2.6		268	10.1	8.9	
Age of children (months)								0.001				0.017
6–11	416	69	16.6	14.9	33	7.9	7.5		102	24.5	22.3	
12–23	800	66	8.3	7.2	25	3.1	2.8		91	11.4	9.9	
24–35	835	52	6.2	5.7	15	1.8	1.2		67	8.0	6.9	
36–47	1114	52	4.7	4.6	54	4.8	4.2		106	9.5	8.8	
48–59	1150	66	5.7	5.8	17	1.5	1.0		83	7.2	6.8	
60–71	914	44	4.8	5.0	13	1.4	1.2		57	6.2	6.2	
Ethnicity of children								0.319				0.610
Han	4665	320	6.9	6.5	128	2.7	2.5		448	9.6	9.1	
Minorities	564	29	5.1	4.6	29	5.1	3.0		58	10.2	7.5	
Birth weight								0.295				0.263
< 2500 g	190	19	10.0	9.3	5	2.6	3.3		24	12.6	12.5	
2500–3999 g	4695	309	6.6	6.1	140	3.0	2.5		449	9.6	8.7	
≥ 4000 g	344	21	6.1	6.2	12	3.5	3.4		33	9.6	9.6	
Preterm birth								0.384				0.187
No	4980	327	6.6	6.2	149	3.0	2.5		476	9.6	8.7	
Yes	249	22	8.8	7.7	8	3.2	4.0		30	12.0	11.7	
Left-behind children								0.415				0.403
No	2257	161	7.1	5.6	67	3.0	2.1		228	10.1	7.7	
Yes	2972	188	6.3	6.8	90	3.1	3.0		278	9.4	9.8	
Feeding practice within 6 months after birth								0.101				0.045
Exclusive breast-feeding	2796	209	7.5	7.4	96	3.4	3.3		305	10.9	10.7	
Predominant breast-feeding	613	36	5.9	5.1	12	2.0	1.3		48	7.8	6.5	
Mixed feeding	1270	72	5.7	5.1	36	2.8	2.4		108	8.5	7.5	
Formula feeding	550	32	5.8	5.5	13	2.4	1.7		45	8.2	7.2	
Nutrient supplements								0.165				0.210
No	2730	157	5.8	5.4	82	3.0	2.5		239	8.8	7.9	
Yes	2499	192	7.7	7.4	75	3.0	2.7		267	10.7	10.1	
Passive smoking								0.276				0.926
No	2885	203	7.0	6.5	84	2.9	2.3		287	9.9	8.8	
Yes	2344	146	6.2	5.9	73	3.1	3.0		219	9.3	8.9	
Regular physical examination								0.036				0.630
No	593	31	5.2	4.5	30	5.1	3.1		61	10.3	7.6	
Yes	4636	318	6.9	6.6	127	2.7	2.5		445	9.6	9.1	
Stunting								0.920				0.846
No	4994	332	6.6	5.8	151	3.0	2.4		483	9.7	8.9	
Yes	235	17	7.2	6.3	6	2.6	2.6		23	9.8	8.2	
Underweight								0.097				0.190
No	5020	329	6.6	6.2	149	3.0	2.5		478	9.5	8.7	
Yes	209	20	9.6	7.2	8	3.8	4.3		28	13.4	11.5	
Wasting								0.666				0.282
No	5016	331	6.6	5.8	150	3.0	2.5		481	9.6	8.8	
Yes	213	18	8.5	6.3	7	3.3	3.6		25	11.7	9.4	
Overweight/obesity								0.258				0.163
No	4631	316	6.8	6.5	135	2.9	2.5		451	9.7	9.1	
Yes	598	33	5.5	4.6	22	3.7	2.6		55	9.2	7.1	
Family income (RMB/year)								0.035				0.005
≤ 30,000	3218	235	7.3	7.2	113	3.5	3.5		348	10.8	10.7	
30,001–59,999	1209	76	6.3	5.6	24	2.0	1.4		100	8.3	7.0	
≥ 60,000	802	38	4.7	4.3	20	2.5	1.5		58	7.2	5.9	
**Characteristics of caregivers**												
Type of caregivers								0.281				0.357
Parents	3431	230	6.7	6.6	114	3.3	3.0		344	10.0	9.6	
Grandparents/ other	1798	119	6.6	5.6	43	2.4	1.9		162	9.0	7.4	
Education level of caregivers								0.033				0.016
Primary school or below	1100	58	5.3	3.7	19	1.7	1.1		77	7.0	4.8	
Junior high school	2013	140	7.0	6.1	73	3.6	2.8		213	10.6	8.9	
High school	1178	81	6.9	7.2	39	3.3	3.2		120	10.2	10.4	
College or above	938	70	7.5	8.9	26	2.8	3.4		96	10.2	12.3	
Occupation of caregivers								0.425				0.511
Housework	3023	196	6.5	5.8	92	3.0	2.3		288	9.5	8.1	
Government agencies staff	612	42	6.9	7.6	10	1.6	2.1		52	8.5	9.7	
Business services staff	435	22	5.1	4.4	14	3.2	2.9		36	8.3	7.3	
Farmer	347	26	7.5	6.2	12	3.5	3.2		38	11.0	9.4	
Other	812	63	7.8	7.7	29	3.6	3.6		92	11.3	11.2	
Anemia-related feeding knowledge level								0.028				0.046
Low	1365	98	7.2	6.2	53	3.9	3.2		151	11.1	9.4	
Moderate	1827	119	6.5	6.5	65	3.6	3.3		184	10.1	9.8	
High	2037	132	6.5	6.1	39	1.9	1.5		171	8.4	7.6	
**Gestational conditions of mothers**												
Maternal moderate/severe anemia								0.008				0.003
No	4980	325	6.5	6.1	143	2.9	2.5		468	9.4	8.6	
Yes	249	24	9.6	8.7	14	5.6	5.2		38	15.3	13.9	
Pregnancy comorbidity								0.156				0.043
No	4743	309	6.5	6.2	137	2.9	2.5		446	9.4	8.7	
Yes	486	40	8.2	7.3	20	4.1	3.8		60	12.3	11.0	
Folate supplementation during pregnancy								0.603				0.267
No	562	34	6.0	5.9	22	3.9	3.2		56	10.0	9.1	
Yes	4667	315	6.7	6.3	135	2.9	2.5		450	9.6	8.8	
Iron supplementation during pregnancy								0.523				0.394
No	1814	109	6.0	5.7	63	3.5	3.1		172	9.5	8.8	
Yes	3415	240	7.0	6.6	94	2.8	2.3		334	9.8	8.9	
Total	5229	349	6.7	6.3	157	3.0	2.6	–	506	9.7	8.8	–

^a^ denotes the crude prevalence calculated by simple samples analyses

^b^ denotes the corrected prevalence calculated by complex samples analyses

^c^ The *P* values calculated by chi-square test of complex samples analyses

### Associated factors of overall anemia and anemia severities in children

Table 5 shows the associated factors of overall anemia and anemia severities among children in multivariate logistic regression analyses. Children age groups of 6–11 months (AOR = 4.71, 95%CI: 2.34–9.47), 12–23 months (AOR = 1.68, 95%CI: 1.17–3.69) and 36–47 months (AOR = 1.59, 95%CI: 1.09–2.83), exclusive breast-feeding within 6 months after birth (AOR = 1.58, 95%CI: 1.04–2.42), and maternal moderate/severe anemia (AOR = 1.77, 95%CI: 1.05–2.98) were significantly associated with an increased risk of overall anemia in children. Children age groups of 6–11 months (AOR = 3.69, 95%CI: 1.61–8.43) and 12–23 months (AOR = 1.78, 95%CI: 1.05–3.16) were significantly associated with an increased risk of mild anemia in children. Children age groups of 6–11 months (AOR = 6.71, 95%CI: 3.02–14.89), 12–23 months (AOR = 2.19, 95%CI: 1.02–4.97) and 36–47 months (AOR = 3.55, 95%CI: 1.89–6.65), low caregivers’ anemia-related feeding knowledge level (AOR = 1.99, 95%CI: 1.18–3.56), and maternal moderate/severe anemia (AOR = 2.33, 95%CI: 1.05–5.18) were significantly associated with an increased risk of moderate/severe anemia in children. Children who underwent regular physical examination were less likely to have moderate/severe anemia, with an AOR of 0.63 (95%CI: 0.41–0.95). The common protective factor for overall, mild and moderate/severe anemia in children was high family income, with AORs of 0.69 (95%CI: 0.50–0.93), 0.75 (95%CI: 0.56–0.99) and 0.51 (95%CI: 0.27–0.98), respectively.

**Table 5:** Associated factors of overall anemia and anemia severities among children in multivariate logistic regression analyses. The values of AOR and 95%CI were calculated by multivariate logistic regression analyses of complex samples

Factors	Mild anemia		Moderate/severe anemia		Overall anemia	
	AOR	95%CI	AOR	95%CI	AOR	95%CI
Age of children (months)						
6–11	3.69	1.61–8.43	6.71	3.02–14.89	4.71	2.34–9.47
12–23	1.78	1.05–3.16	2.19	1.02–4.97	1.68	1.17–3.69
24–35	1.15	0.62–2.16	0.96	0.38–2.42	1.13	0.64–1.99
36–47	0.99	0.49–1.99	3.55	1.89–6.65	1.59	1.09–2.83
48–59	1.18	0.70–1.99	0.78	0.26–2.33	1.08	0.65–1.81
60–71	1.00		1.00		1.00	
Feeding practice within 6 months after birth						
Exclusive breast-feeding					1.58	1.04–2.42
Predominant breast-feeding					0.93	0.58–1.50
Mixed feeding					0.98	0.51–1.88
Formula feeding					1.00	
Regular physical examination	1.44	0.80–2.61	0.63	0.41–0.95		
Family income	0.75	0.56–0.99	0.51	0.27–0.98	0.69	0.50–0.93
Anemia-related feeding knowledge level						
Low	0.99	0.77–1.26	1.99	1.18–3.56		
Moderate	1.03	0.66–1.61	2.06	0.88–4.82		
High						
Maternal moderate/severe anemia	1.46	0.81–2.62	2.33	1.05–5.18	1.77	1.05–2.98

*AOR* adjusted odds ratio, *CI* confidence interval

## Discussion

### Anemia status of children

The finding of this study showed that the overall anemia prevalence among children aged 6–71 months in rural Hunan Province was 8.8%, which was significantly lower than the preschool children in Africa (50.4–70.9%) or Latin America and Caribbean (32.9%) [28–30], but was slightly higher than the children aged 6–59 months in America in 2016 (8.6%) [31]. Compared with the 2010 national epidemiological data in China, the anemia status of children was significantly improved and the anemia prevalence declined from 13.3 to 8.8%, with a declining amplitude of 34% [2]. According to the WHO anemia public health classification, the anemia status of preschool children in rural Hunan was classified as a mild public health problem and was basically below the level of children anemia control (< 12%) set by *China Child Development Outline (2011–2020)*.

The possible reason for the low anemia prevalence in this study area may be that the Chinese government has given much investment and attention to the nutrition improvement of children in poor rural areas in the past decade. The Chinese government has implemented children nutrition improvement projects in poor rural areas throughout 100 counties of 10 provinces, including Hunan, since 2012, and expanded the coverage to 341 counties in 21 provinces since 2014. Specifically, children aged 6–23 months in poor rural areas are provided for free with 1 bag/day of complementary food supplement (Yingyangbao for short) containing 6 vitamins (vitamin A, B_1_, B_2_, B_12_, D_3_, folate) and 3 minerals (iron, zinc, calcium), and childhood nutrition knowledge (including scientific feeding, malnutrition and anemia prevention) is propagandized to children’s caregivers through various forms of diffusion campaigns in the project [16]. This project covered 20 counties in Hunan at the beginning and expanded to 25 counties in 2014 and to 53 counties in 2018. At this stage, all poor rural areas in Hunan were covered, and the annual accumulated number was 287,000 targeted children. Among the children over 2 years old investigated in this study, 86.0% (3452/4013) had taken Yingyangbao when they were 6–23 months old; and the proportion of Yingyangbao for in children aged 6–23 months was 91.7% (1115/1216). Many studies showed that Yingyangbao intervention and nutrition education could significantly improve the diet quality and nutritional status of children, and good compliance to Yingyangbao contributed to a low risk for childhood anemia [32, 33].

In our study, the anemia severity was mainly mild, followed by moderate, which was in agreement with similar studies conducted in Ethiopia [34] and Haiti [35]. There were two possible reasons for the fact. On the one hand, children with mild anemia were mostly asymptomatic and thus did not attract enough attention from their caregivers, so the caregivers might not seek medical intervention and did not give the children prompt and effective treatment [34]. On the other hand, childhood anemia was nutritional anemia in most cases and was dominated by mild iron-deficiency anemia.

### Associated factors on anemia

#### Age of children

Many of the published observational studies have confirmed that child age is a key determinant factor on anemia [11, 13, 29, 34, 36, 37]. An epidemiological investigation in Iran reported that the older age of children was a protective factor for childhood anemia, and the risk of anemia decreased by 12% with the rise of 1 month of age [36]. A cross-sectional study conducted by Endidaye in Ethiopia found that the risks of anemia in age groups of 6–11 months and 12–23 months were 5.67 and 5.80 times of the age group of 48–59 months, respectively [37]. The association of child age with anemia in our study was consistent with the above researches, found that as the child getting older, the overall anemia prevalence decreased from 22.3% (in the age group of 6–11 months) to 6.2% (in the age group of 60–71 months) which meant that children at younger age were the most vulnerable group for anemia. The likelihood of being anemic for the children in the age group of 6–11, 12–23 and 36–47 months was 4.71, 1.68 and 1.59 times of the age group of 60–71 months. Like the overall anemia prevalence, age group was also associated with the anemia severity, as younger age was a risk factor of both mild anemia and moderate/severe anemia. Since the age group of 6–23 months was the key period for complementary food introduction and eating habits nurturance, inappropriate complementary food introduction or poor eating habits in this age group would easily cause insufficient intake of iron nutrient and led to iron-deficiency anemia [7, 14, 34]. Moreover, in China, when children reached 36 months of age, they could go to kindergartens for collective life, but some kindergartens provided low-quality nutritious diets that did not meet the demands of children for normal growth and development and consequently, the risks of anemia at the age group of 36–47 months was increased [38].

### Feeding practice

As reported, feeding practice was closely associated with childhood anemia, and breast-feeding was at increased risk of childhood anemia compared to formula feeding [10, 17, 39, 40]. An observational study conducted in China showed that compared with formula feeding, both exclusive breast-feeding and mixed feeding increased the risks of iron deficiency and iron-deficiency anemia among infants aged 9 months [39]. A cross-sectional study conducted in South Korea also found that infants aged 8–15 months fed only with or mainly with breast milk might be more prone to iron deficiency and iron-deficient anemia [40]. Our study showed that exclusive breast-feeding within 6 months after birth increased the risk of anemia by 58% compared with formula feeding, but we did not find any association between predominant breast-feeding or mixed feeding with anemia in children. The possible explanation was that the iron stored in infants’ body would be depleted within 4 months after birth, and the iron concentration in breast milk was low, and despite the high bioavailability, the iron in breast milk did not meet the demand for growth and development by infants. A cohort study involving 270 infants (followed up from birth to 6–8 months after birth) from Bolivia confirmed this viewpoint that the hemoglobin and serum ferritin concentrations significantly dropped with the prolonged time of exclusive breast-feeding [10].

Exclusive breast-feeding as the recommended feeding practice for infants within 6 months after birth by WHO was indisputably beneficial to infants and young children, and was outstanding with complete nutrition composition and immunodominance, that was incomparable by other ways of feeding. Hence, *2012 Children’s Nutritional Disease Management Technical Specification* issued by China Ministry of Health definitely provided that term infants depending on exclusive or predominant breast-feeding should be supplemented with iron since the 4th month after birth, and the daily intake should be 1 mg/kg elemental iron, which would prevent the occurrence of iron-deficient anemia.

### Maternal anemia

Many of the published papers have confirmed that maternal anemia can induce inadequate iron store in infants and is more likely to cause iron-deficient anemia in infants after birth [9, 37, 41]. A cohort study from India found the hemoglobin and serum ferritin levels in infants born to anemic mothers were significantly lower than those born to non-anemic mothers both at birth and 14 weeks after birth [41]. An observational study in Southern Africa reported that maternal anemia raised the risk of anemia in children aged 6–59 months by 52–71% [9]. In the present study, maternal moderate/severe anemia was an independent risk factor of childhood anemia and was significantly associated with childhood moderate/severe anemia. Compared with children born to mothers without moderate/severe anemia, the risk of moderate/severe anemia in children born to mothers with moderate/severe anemia were increased by 133%, and the overall anemia risk rose by 77%. Hence, prenatal care and gestational nutrition of mothers and prompt addressing of maternal anemia are significant for anemic prevention in children.

### Preterm birth and low birth weight

Like maternal anemia during pregnancy, preterm birth and low birth weight also can lead to inadequate fetal iron store, which makes preterm infants and low-birth-weight infants more susceptible to anemia. A cross-sectional study involving 1127 infants aged 6 months showed the anemia prevalence of preterm infants was significantly higher than that of term infants (38.5% vs. 10.2%) [5]. An observational study conducted in Switzerland reported that low birth weight raised the risk of anemia in infants aged 6–23 months by 16% [8]. Our study found no association between preterm birth or low birth weight with childhood anemia. This might be due the fact that the preterm infants or low-birth-weight infants in China were routinely supplemented with iron (2 mg/kg/day) since the 4-th week after birth until 1 year old, which efficiently compensated for the inadequate iron store due to preterm birth or low birth weight and thereby prevented iron-deficient anemia in preterm infants and low-birth-weight infants.

### Nutritional status of children

Several published studies have confirmed that childhood malnutrition is closely related to anemia [7, 14, 42]. Malnutrition often coexists with other micronutrient deficiencies (e.g. iron, zinc, folate, vitamin A, vitamin B_12_), which may increase the development of anemia by a synergistic association. A cross-sectional study carried out in 2012 in northwestern China showed that stunting, underweight, and wasting all were risk factors for suffering from anemia, with AORs of 1.65, 2.42 and 2.89, respectively [7]. Unlike previously published results [7, 14, 42], our study found no association between malnutrition with childhood anemia. This inconsistency might be due to following explanations. The rates of stunting, underweight, and wasting of children were relatively low in our study, all of which were less than 5%; and each type of malnutrition was mainly moderate, with very little severity, so the relationship between malnutrition and anemia had not been discovered in our study.

### Regular physical examination and caregivers’ anemia-related feeding knowledge

This study found out that regular physical examination was a protective factor for moderate/severe anemia in children, but found no association between regular physical examination and mild anemia in children. The possible reason for this association might be that children with moderate/severe anemia had obvious clinical symptoms and were more likely to pursue medical interventions and thus would be effectively treated in time. In addition, our study found that the lower caregivers’ anemia-related feeding knowledge level was a risk factor of moderate/severe anemia in children, which was consistent with a study conducted in Indonesia [43]. As reported, maternal knowledge of anemia promoted the development of some health behaviors related to anemia reduction (e.g. rational diets, regular physical examination, preventive iron agent supplementation), which would decrease the occurrence of anemia in children [43]. In the present study, the caregivers’ anemia-related feeding knowledge level largely decided the diet quality of children, while the diet quality was closely related with anemia in children. Two observational studies from Ethiopia also showed that poor diet would greatly increase the risk of childhood anemia [11, 44].

### Family income

High family income was also identified as a protective factor for childhood anemia, which was supported by previous studies [7, 11, 13]. The data from the 2011 Bangladesh Demographic Health Survey showed that the socioeconomic status was closely related to childhood anemia, preschool children from low- and middle-income families were more likely to be anemic compared with their counterparts from high-income families [45]. Our results found that as family income increased, the risk of anemia in children decreased, which was consistent with previous similar studies. Families with high incomes have the ability to purchase and provide good nutritious foods, so the dietary patterns for their children are more reasonable, which decreases the occurrence of anemia.

### Limitations

This study has several limitations. First, owing to the inherent characteristics of cross-sectional studies, the relationship between investigated factors and anemia identified in our study was statistical association, rather than causality. Second, the data collection about maternal anemia status and feeding practices within 6 months after birth were both based on the recalling of past event by the caregivers, which unavoidably led to recall bias. Third, anemia diagnosis was based on hemoglobin levels alone, but no etiological diagnosis, which limited the classification of subtypes of anemia. Fourth, there might be potential blood sample collection errors and HemoCue301 device measurement errors in the hemoglobin detection. The accuracy of hemoglobin measurement was closely related to the specimen collection and hemoglobin analyzer operation by the operators. The accuracy of hemoglobin measurement could be affected by the excessive compression at the blood sampling site, insufficient blood absorption on blood spots, bubble generation, or mistaken scraping of blood on the spots while wiping excessive blood. Finally, certain important factors were unavailable in our study, such as complementary food introduction, dietary patterns, parasitic infections, and genetic hemoglobin disorders. Therefore, these factors were not included in the final analysis. Nevertheless, this large-size epidemiological survey involved 5229 children from 72 villages across 24 towns in 12 counties of Hunan Province, and the investigated ages covered the whole preschool age range. Hence, our findings reflected the current anemia status and associated factors of preschool children in rural Hunan and would help health administrative departments to take measures to reduce the anemia-induced burdens in rural areas.

## Conclusions

The anemia status of preschool children in rural Hunan Province was a mild public health problem and associated with children age group, feeding practice, regular physical examination, family income, caregivers’ anemia-related feeding knowledge level, and maternal moderate/severe anemia. The key population for childhood anemia prevention and control in rural areas is children aged 6–23 months. Exclusive breast-feeding should be encouraged for infants within 6 months after birth, but attention should be paid to preventive iron agent supplementation. While strengthen the health education for caregivers’ scientific feeding knowledge, the prevention and treatment of maternal anemia during pregnancy needs to be further improved.

## Supplementary information

**Additional file 1.** The corrected values of hemoglobin at different altitudes.

## Data Availability

The datasets generated during and/or analysed during the current study are not publicly available duo to the personal privacy of subjects but are available from the corresponding author on reasonable request.

## Abbreviations

WHO: World Health Organization
Hb: Hemoglobin
OR: Odds Ratio
CI: Confidence Interval
