# Supplementary material for: Anemia prevalence, severity and associated factors among children aged 6–71 months in rural Hunan Province, China: a community-based cross-sectional study
Source: BMC Public Health. 2020 Jun 23;20:989. doi: 10.1186/s12889-020-09129-y (PMC7310416; doi:10.1186/s12889-020-09129-y)
Supplement: Supplementary file 1 — Additional file 1. The corrected values of hemoglobin at different altitudes. [file 12889_2020_9129_MOESM1_ESM.docx]

Additional file 1

**Additional file 1** The corrected values of hemoglobin at different altitudes.

| Altitude, meter | Corrected value of hemoglobin, g/L |
| --- | --- |
| <1000 | +0 |
| 1000- | +2 |
| 1500- | +5 |
| 2000- | +8 |
| 2500- | +13 |
| 3000- | +19 |
| 3500- | +27 |
| 4000- | +35 |
| 4500- | +45 |
